# 3D Bioprinting Technologies for Hard Tissue and Organ Engineering

**DOI:** 10.3390/ma9100802

**Published:** 2016-09-27

**Authors:** Xiaohong Wang, Qiang Ao, Xiaohong Tian, Jun Fan, Yujun Wei, Weijian Hou, Hao Tong, Shuling Bai

**Affiliations:** 1 Department of Tissue Engineering, Center of 3D Printing Organ Manufacturing, School of Fundamental Sciences, China Medical University (CMU), No. 77 Puhe Road, Shenyang North New Area, Shenyang 110122, China; aoqiang00@163.com (Q.A.); xhtian@cmu.edu.cn (X.T.); jfan@cmu.edu.cn (J.F.); Weiyj2011@gmail.com (Y.W.); wjhou@cmu.edu.cn (W.H.); tongh007@hotmail.com (H.T.); baishuling@hotmail.com (S.B.); 2 Department of Mechanical Engineering, Tsinghua University, Center of Organ Manufacturing, Beijing 100084, China

**Keywords:** hard tissues and organs, mechanical properties, composite materials, bones, teeth, cartilage

## Abstract

Hard tissues and organs, including the bones, teeth and cartilage, are the most extensively exploited and rapidly developed areas in regenerative medicine field. One prominent character of hard tissues and organs is that their extracellular matrices mineralize to withstand weight and pressure. Over the last two decades, a wide variety of 3D printing technologies have been adapted to hard tissue and organ engineering. These 3D printing technologies have been defined as 3D bioprinting. Especially for hard organ regeneration, a series of new theories, strategies and protocols have been proposed. Some of the technologies have been applied in medical therapies with some successes. Each of the technologies has pros and cons in hard tissue and organ engineering. In this review, we summarize the advantages and disadvantages of the historical available innovative 3D bioprinting technologies for used as special tools for hard tissue and organ engineering.

## 1. Introduction

Hard tissues and organs in the human body include the bones, teeth and cartilage, consisting of certain unique cell types and substantial organic and inorganic extracellular matrices (ECMs). For example, the bone is composed of osteoblasts and calcified ECMs, in which the majority inorganic ECM is hydroxyapatite (HA). The tooth is another highly calcified hard tissue. It consists of the enamel, cementum, dentin and endodontium [1]. Whereas the cartilage includes articular gristle, and the main constitutes of noses and ears [2]. These hard tissues and organs take the role of mechanical support with some basic biological functions, such as hematopoiesis and metabolism, which are vitally important in maintaining human lives and activities [3]. 

Hard tissue and organ defects, such as bone tumor, tooth fall and ear deformity, have caused tremendous harms to people’s health status and life quality. Generally, the small defects can be cured through host tissue/organ self-regeneration. However, the large defects (e.g., ≥1 cm in length) need intervention therapies, such as implanting grafts to promote healing or repair [4,5,6,7,8,9]. Traditionally, autologous tissue has been considered as gold standard for bridging large hard tissue defects after accidents or cancer surgery. However, the use of autologous tissue always encounters the risks of a second operation after the implantation with some unexpected syndromes. Clinically, there is a great need for novel, stable and resorbable large hard tissue and organ repair materials that are made by 3D printing technologies [10,11]. 

The production of hard tissue and organ substitutes (also named as implants, grafts, biomaterials, prostheses, precursors and analogues) is an important part of regenerative medicine. Among which, the fabrication of bone repair materials has started earlier and the clinical applications are more successful [4,5,6,7,8,9,10,11]. A special need of the hard tissue and organ substitutes is that they require high content of inorganic ECMs with strong mechanical properties. So, for hard tissue and organ engineering, the material constitutes and structural characteristics of the substitutes have always been the research focuses. Particularly, biomaterials, which have been used frequently as hard tissue and organ implants, have undergone several development stages, such as passive commercial products, no bioactive scaffolds, cell-laden hydrogels, and pre-designed initiative smart composites [12,13,14,15,16]. Additionally, some hard organs, such as the nose and ears, have complex curved surfaces which require specific processing technologies to manufacture. Therefore, the development of new hard tissue and organ substitutes with suitable physical and biological functions based on the bionic principles is an important area of hard tissue and organ engineering [17,18,19,20,21,22,23]. 

3D printing, also named as solid freeform fabrication (SFF), additive manufacturing (AM), layered manufacturing (LM) or rapid prototyping (RP), is a family of enabling technologies that can produce solid objects layer-by-layer using computer aided design (CAD) models [24,25]. Compared with traditional tissue engineering approaches, 3D printing technologies are often sophisticated, flexible, and automated [26,27,28]. Through the use of 3D printers, the manufacturing procedures can be dramatically simplified. Over the last decade, many industrial 3D printers have been employed to generate porous scaffolds for hard tissue engineering [29]. Whereas some distinctive 3D printers for cell-laden tissue and organ manufacturing have drastically increased [12,13,14,15,16,17,18,19,20,21,22,23,26,27,28]. The 3D printing technologies have been already described as the third industrial revolution with number of new publications increasing rapidly [30]. 

**Figure 1:**
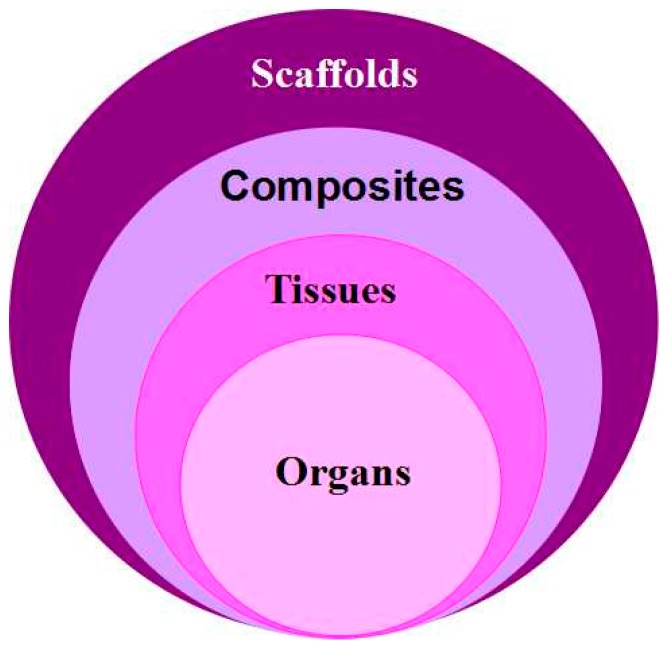
Applications of 3D printing technologies in regenerative medicine: the produced 3D objects can be porous scaffolds, cell/biomaterials composites, homogeneous tissues, or multiple tissues contained organs.

The main advantage of 3D printing technologies in large hard tissue and organ engineering is their capability to produce complex 3D objects rapidly from a computer model with varying internal and external structures, such as go-through channels. These complex 3D objects can be either tissue engineering porous scaffolds, cell/biomaterial composites, homogeneous tissues, or multiple tissue contained organs (Figure 1). After printing, the porous 3D scaffolds can be implanted alone or seeded with autologous cells to serve as osteoconductive templates in large tissue engineering. Ideally, new tissue forms along the go-through channels during the scaffolds degrade slowly in the body [31,32]. The cell/biomaterial composites can be used in vitro or in vivo for large hard tissue regenerative research. The homogeneous tissues can be used for large hard tissue defect repair. While the multiple tissue contained organs can be used for customized organ engineering and substitution. Currently, there is a wide range of materials can be used for the 3D printing processes. 

Currently, there is a wide range of materials which have been used for the 3D printing processes. For example, 3D printed metal hip joints are considerably lighter than the ones produced by conventional methods. With the go-through channels, the implants can remain longer in the body than conventional implants due to the coalescence of the 3D printed implants with the host bones. Hard tissues can grow easily into the go-through channels and enhance the repair effects. Subsequently, synthetic polymer based scaffolds with similar material properties as natural real bones have been extensively researched. One of the advantages of these synthetic scaffolds is that they—unlike metal implants—behave neutrally in X-ray equipment [33,34]. It is now possible to reconstruct an outline of an ear or a jaw that exactly mimicks the patients’ large tissue and organ contours based on the images acquired by magnetic resonance imaging (MRI) or computerized tomography (CT) scans directly from the patients. The predefined go-through channels have a direct impact on the outcomes of the hard tissue and organ repairs [35].

During the last three decades, various metal implants have become the main solutions for large hip replacement and long bone graft. Some metal powders have been used for 3D printing. Theses metal powders include titanium, stainless steel, tantalum, aluminum alloys, Inconel, nickel-based alloys, titanium aluminides, and their composites. Xue et al. have employed 3D techniques to make titanium scaffolds with an average pore size of 800 µm and porosity of 17%–58% [36]. This porous titanium scaffold improved the clinical performance of the metal substitutes by promoting osteoblasts to adhere and proliferate inside. When the titanium scaffold was implanted into the target location, osteoblasts migrated into the go-through channels, proliferated and secreted ECMs, leading to the reconstruction of the damaged bone along the gradually degraded metal scaffold. However, metal implants can cause many vice reactions or syndromes for hard tissue and organ regeneration. 

As stated above, hard tissues and organs have unique material and structural characteristics that give them their strength. An advantage of 3D printing over traditional tissue engineering strategies is the ability of 3D printing to include these material and structural elements in the fabrication processes of the hard tissue and organ analogues. Especially, many hard organs have soft tissues (such as bone marrow in the bones and pulp in the teeth) that are hard to fabricate using traditional tissue engineering approaches. In this review, we summarized some of the innovative 3D printing technologies for hard tissue and organ engineering obtained over the last three decades with emphasis on functional aspect of each technology, suitable printing materials, strengths and weaknesses in hard tissue and organ engineering.

## 2. 3D Printing Technologies

First developed in the 1980s, 3D printing refers to many different methods of creating original looking objects from CAD files [37]. The printing principles can be imagined as placing a certain number of coastal layers onto each other to build up a coaster cube (i.e., 3D object) [38]. Digital manufacturing serves as a general term for computer-aided production and includes various technical procedures. A processed digital model (e.g., CAD file) is always employed. With the rapid development of this area, a series of advanced processing technologies have been applied to hard tissue and organ engineering [26,27,28,29,30].

### 2.1. Classification of 3D Printing Technologies

3D printing technologies can be classified in several different ways based on the working principles, pre-material (base material or starting material) states, energy sources and biological functions of the products. 

#### 2.1.1. Categories Divided in Working Principles

3D printing technologies can be divided into seven main groups according to the working principles used to produce 3D objects: (1) binder jetting RP (also known as powder bed and inkjet head 3D printing) is a process in which a liquid bonding agent (such as, polymer solution) is selectively deposited in conjunction with powder materials [21,39]; (2) material extrusion RP, such as fused deposition modeling (FDM)/fused filament fabrication (FFF) and stick deposition molding (SDM), is a process in which material is selectively dispensed through a nozzle or orifice [21,39]; (3) directed energy deposition RP, such as electron beam direct manufacturing (EBDM) and laser powder forming (LPF), is a process in which focused thermal energy (e.g., laser, ultraviolet (UV), electron beam and plasma arc) is used to fuse or melt the materials being deposited [21,39]; (4) powder based fusion RP, such as selective laser sintering (SLS), selective laser melting (SLM), selective heat sintering (SHS), and electron beam melting (EBM), is a process in which thermal energy is used to selectively fuse regions of a powder bed [21,39]; (5) material jetting RP, such as multiJet printing (MJP)/multiJet modeling (MJM), polyJet printing, and contour crafting (CC), is a process in which droplets of build material are selectively deposited [21,39]; (6) vatphotopolymerization RP, such as stereolithography (SLA or SL), digital light processing (DLP), and scan-LED technology (SLT), is a process in which liquid photopolymer in a vat is selectively cured by light-activated polymerization [21,39]; and (7) sheet lamination RP, such as laminated object modeling (LOM), and film transfer imaging (FTI) or selective deposition lamination (SDL), is a process in which sheets of material are bonded to form an object [21,39]. Most of these 3D printing technologies, such as binder jetting, FDM/FFF, SDM, EBDM, LPF, SLS, SLM, SHS, EBM, MJP/MJM, CC, SLA/SL, DLP, SLT, LOM, FTI and SDL, are initially used for metal, paper and plastic material reshaping. 

#### 2.1.2. Categories Divided in Starting Material States

3D printing technologies can be divided into the following three main procedures according to the base (or starting) material states: (1) fluid material RP technologies; (2) powder material RP technologies; and (3) solid material RP technologies. Each of the groups has many subgroups, such as SLA, MJP, polyJet printing, solid object ultraviolet-laser printing, 3D bioprinting, rapid freeze prototyping, and bioplottering for fluid material RP technologies; SLS, colorJet printing (CJP), EBM, SLM, and EOSINT systems for powder material RP technologies; FDM/FFF, SDL, LOM and ultrasonic consolidation for solid material RP technologies. Among these 3D printing technologies, SLA, MJP, SLS, and SLM are currently the main procedures in hard tissue scaffold manufacturing with the addition of inorganic materials, such as HA and calcium phosphate.

#### 2.1.3. 3D Printing Categories in Energy Sources

**Figure 2:**
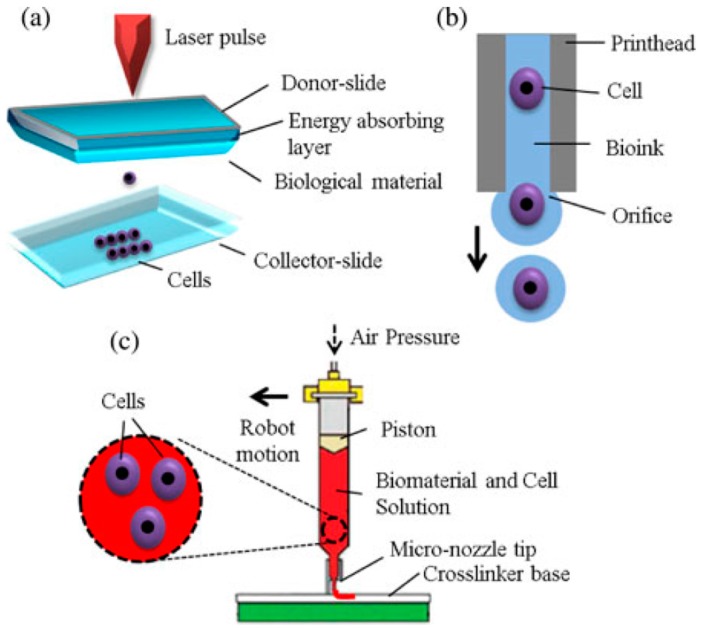
Working principles of three main groups of bioprinting technologies for tissue and organ engineering: (**a**) laser-based bioprinting; (**b**) inkjet-based bioprinting; and (**c**) extrusion-based bioprinting [22].

In addition, 3D printing technologies can be divided into the following six main groups according to the energy sources: (1) inkjet-based printing; (2) laser-based printing; (3) force (extrusion)-based printing; (4) ultrasonic-based printing; (5) electron beam-based printing; and (6) UV-based printing. Each group has a large family. For example, powder metal deposition, laser consolidation (LC), laser metal forming (LMF) and laser engineered net shaping (LENS) all belong to the laser-based 3D printing group. Among the above six groups, the first three groups have been widely used in hard tissue and organ engineering. Especially, some porous metal scaffolds have been applied clinically as biodegradable or non-degradable hard tissue engineering templates. The working principles of the inkjet-, laser-, and extrusion-based bioprinting technologies are summarized in Figure 2 [22]. 

#### 2.1.4. Categories Divided in Biological Functions

3D printing technologies can also be divided into the following two groups according to the biological functions of the products: (1) printing without living cells; and (2) printing with living cells [21]. The printing requirements for each group are very different. For example, when printing without living cells, the main requirements for the 3D printing technologies are the accuracy of the scaffold structures, the stability of the connected layers, the flexibility of the go-through pores and the biocompatibility of the deposited materials. When printing with living cells, the main requirements for the 3D printing technologies are the viability of the cells, the growth capacity of the tissues and the biological functionality of the implants. The latter has been defined as 3D bioprinting by tissue engineers. Thus, 3D bioprinting is the process of creating cell patterns in a confined space using 3D printing technologies, where cell function and viability are preserved within the printed construct [40,41]. We now would like to introduce the following three major types (i.e., inkjet-based, laser-based and extrusion-based) of 3D bioprinting technologies.

### 2.2. Three Main 3D Bioprinting Technologies

#### 2.2.1. Inkjet-Based 3D Bioprinting

**Figure 3:**
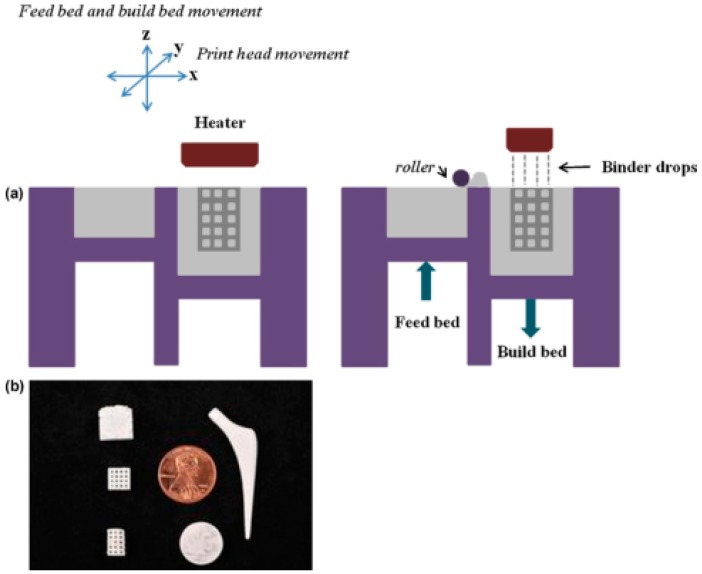
(**a**) 3D printing schematic using an inkjet printing system; and (**b**) 3D printed calcium phosphate (CaP) sintered structures fabricated at Washington State University using a 3D printer (ProMetal^®^, ExOne LLC, Irwin, PA, USA) [42].

Inkjet-based 3D bioprinting is a non-contact image reconstruction technology (Figure 3), which includes piezoelectric, thermal and acoustic conductivity nozzles. Normally, inkjet 3D bioprinting techniques are derived directly from commercially available 2D printers and employ ink binding starting materials, such as polymer solutions, to form desired objects [42,43]. Inkjet printers usually consist of one or several ink chambers with different nozzles corresponding to piezoelectric, thermal, or acoustic actuating units. A short pulse of electrical current is needed to actuate the units. Before printing, the starting materials need to be liquefied to permit droplets deposition onto a solid platform. During the printing process, a fixed volume of fluid is continually jetted onto the platform through the thermal, acoustic or piezoelectric actuating units and the pre-designed signals reappear on the platform through the ink droplets. The droplets must be solidified into the pre-defined geometry before the next layer of droplets is added. The deposited droplet size can be modulated from 1 to 300 pL with deposition rates changing from 1 to 10,000 droplets per second. Cells are normally printed in suspensions or low concentration polymer solutions.

The advantages of inkjet based bioprinting technologies in hard tissue and organ engineering are fast, cheap, readily available and high resolution. The deposition resolution can be adjusted to about the size of one cell (≈10 µm) and the printing accuracy can be tailored to less than 100 µm [44]. There are several disadvantages of the inkjet bioprinting technologies: (1) the starting materials need to be dissolved into liquid states at low viscosities; (2) the heat, ultrasound, and mechanical stresses (especially shear forces) generated during the inkjet bioprinting have adverse effects on cell viability; (3) it is difficult to update the required hardware and software for multiple cell type assemblings; (4) limited biomaterials used for cell loading because of nozzle (or head) clogging; (5) only low cell numbers can be printed; and (6) finite printing height. Future work needs to be done to develop multi-head printers with heterogeneous cell constitutes and gradient structural information [45,46,47].

#### 2.2.2. Laser-Based 3D Bioprinting

**Figure 4:**
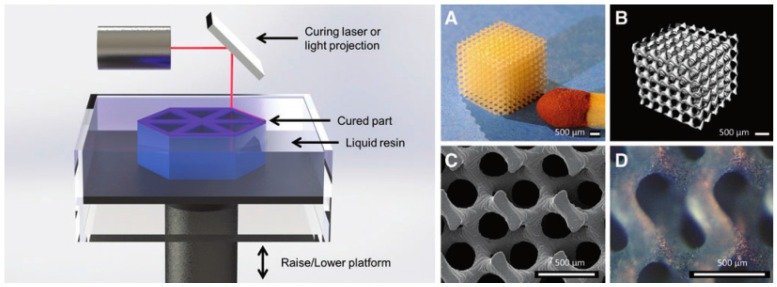
Schematic of stereolithographic (SLA) printing technique; and (**A**–**D**) exemplary tissue engineering scaffold composed of poly(d-l lactic acid) (PDLLA) that showcases the resolution and detail of SLA [47]: (**A**) photograph; (**B**) micro computed tomography (mCT); and (**C**,**D**) scanning electron microscope (SEM). Scale bar is 500 mm.

Laser-based 3D bioprinting technologies are a group of printing methods that use laser energy to transfer or coordinate starting biomaterials (Figure 4). There are many different forms of laser-based 3D bioprinting technologies in hard tissue and organ engineering. For example, laser direct writing (LDW) uses a laser pulse to locally heat and deposit a layer of energy-absorbing starting biomaterial. The starting biomaterials can be cell-laden polymer hydrogels or solutions. Multiple cell types can be simultaneously deposited onto the surface of a work piece. An existing example is that in 2000 Odde and Renn first reported a cell printing technology via a laser-guided direct cell writing method [48,49]. Additionally, these techniques can be further divided into direct RP or indirect RP 3D bioprinting technologies for hard tissue and organ engineering. 

Typically, this group of 3D bioprinting technologies is nozzle free high precision methods for cell patterning [48,49]. Single cells or cell suspensions can be placed onto a platform in a controlled manner. A wide range of viscosities of cell-laden polymer solutions with high cell number can be printed [50,51,52,53,54,55]. Nonetheless, most of these 3D bioprinting technologies have extremely high restrictions on the types of the polymer solutions. It is a time-consuming process for large tissue and organ printing applications. Three more prominent limitations of these techniques are the damages of the laser to cells, cell distributing accurate and metal contaminants. This is why, sixteen years later, this group of 3D bioprinting technologies is still limited to some simple constructs arranged with a thin layer of cells [56]. 

#### 2.2.3. Extrusion-Based 3D Bioprinting

**Figure 5:**
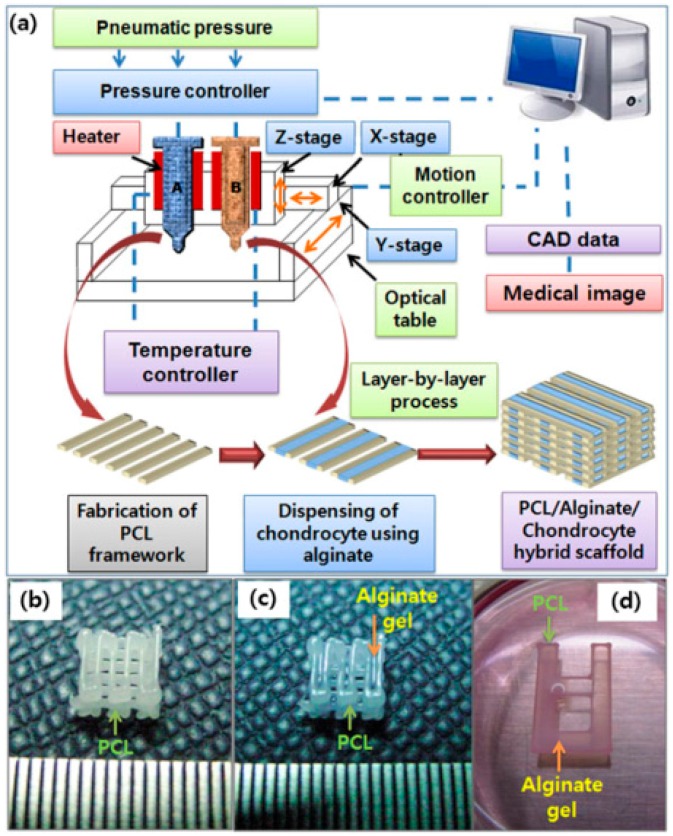
(**a**) Schematics of the fabrication process of cell-printed 3D polycaprolactone (PCL)–alginate gel hybrid scaffold using a multihead deposition system; Photo-images of: (**b**) fabricated porous 3D PCL scaffold; (**c**) chondrocyte-printed 3D PCL–alginate gel hybrid scaffold for in vivo experiments; and (**d**) simplified 2D hybrid scaffold for in vitro experiments [57].

Extrusion-based 3D bioprinting technologies are a swarm of processes in which starting materials are totally dispensed by force through a nozzle, syringe or orifice (Figure 5). There are three broad categories of this group of 3D bioprinting technologies according to the printing temperature (i.e., high-, ambient- and low-temperature). One of the most popular processes is melting extrusion with a very high working temperature for starting material melting, such as fused deposition modeling (FDM) [57,58,59,60]. Some specific plastics, such as acrylonitrile-butadiene-styrene (ABS) and poly(lactice acid) (PLA) that melting about 200 °C, are the most suitable printing materials as nonbiodegadable hard tissue and organ engineering scaffolds. Currently, it is one of the least expensive methods to create solid 3D scaffolds with go-through channels. Other popular processes are ambient- and low-temperature deposition RP manufacturing technologies, which were first put forward by the Center of Organ Manufacturing, Department of Mechanical Engineering, in Tsinghua University and adapted by other labs over the world [61,62,63,64,65,66,67,68]. 

In this research group, headed by Professor Wang, cells were first encapsulated into hydrogels for bioprinting [69,70,71,72,73,74,75]. Natural polymer hydrogels mimic ECMs to provide the cells with suitable conditions to migrate, grow, proliferate and differentiate. The hydrogel concentration and cell density have significant effects on tissue and organ formation and maturation. Many ingredients, such as polymers, growth factors, cryoprotectants, can be added into the natural polymer hydrogels. Using appropriate polymer concentrations, oxygen and nutrients can maximally diffuse into the encapsulated cells. The temperature of the working platform, nozzle and environment can be controlled, which allows a wide range of biomaterials to be printed. Extremely high cell densities and viabilities have been achieved. Because of the advantages of these two groups of 3D bioprinting technologies, implants for patient-specific (or customized) hard tissue and organ regeneration are now available and become more and more attractive.

Compared to inkjet-based and laser-based 3D bioprinting technologies, the printing speed of the extrusion-based 3D bioprinting technologies is relatively slow. Cells encapsulated in the high concentrations natural hydrogels may lose some functions, such as, cell–cell direct interactions or communications. Nevertheless, with the proper concentration of natural hydrogels, cells have enough space to grow, proliferate, and differentiate. The 3D printed construct can mimic the native cell survival environment, recapitulating the in vivo milieu and allowing cells to create their own micro-environments. Furthermore, Additionally, the high capacity of the starting materials and the easy of updating hard- and software make this group of 3D bioprinting technologies outstanding for hard tissue and organ engineering. 

## 3. Examples of 3D Bioprinting Technologies for Hard Tissue and Organ Engineering 

### 3.1. Hard Tissue Scaffolds Printing 

In the hospitals, 3D printing technologies were originally used for the production of visual models and functional prototypes, now they are increasingly employed in the manufacture of hard tissue engineering scaffolds. Nearly all the cell-free 3D printing products, including metal, synthetic and natural polymers, have been used as the hard tissue engineering scaffolds. Metal and HA powders are the frequently used starting materials to enhance the mechanical strength of the hard tissue repair substitutes [11,55]. Additionally, metallic systems can be biodegraded slowly in vivo. The degraded elements, such as iron and manganese ions, can be absorbed in biosystems and act as important minerals for new tissue growth and bone remodeling. This is a new theory for tissue engineering approaches based on seeding cells on porous biodegradable polymer scaffolds.

As the main component of bone, HA has some prominent merits for the use as a pre-material for hard tissue scaffold printing. HA can be produced synthetically or from bovine sponges or by coral pyrolysis and sintering processes. These systems provide an abundant resource. Some of the natural HA particles have good biocompatibilities and high osteoconductivity. In 3D printing technologies HA can be used in different forms, such as powder, slurry or granule. To obtain the fluidity necessary for the 3D printing processes, HA can be modified by means of granulation or mixed with other polymer solutions [76]. A polymer solution is often used as a liquid binder for the coalescent of the powdered HA particles and even the incorporation of cells.

One example is in polymer–ceramic binder jetting 3D printing, HA objects can be obtained by selectively spraying liquid organic binder onto a bed of HA powder and solidifying the powder into a cross-section [77,78]. Many thin layers of HA powder are continuously applied to a base platform (or plate), which are then solidified by adding the specific liquid organic binder according to the predefined pattern. The liquid organic binder can be applied by dribs and drabs using a print head. After printing, the loose HA powder is removed and the solid HA objects are directly used as the hard tissue engineering scaffolds. In some of the established 3D printing processes the solid HA objects can be further sintered in the second step at a temperature of about 1250 °C [55]. This produces high final strength to the 3D objects. During the sintering process, the liquid organic binder is completely burned [79,80].

In 1994, Gima et al. made a hard tissue engineering scaffold using the binder jetting 3D printing process [81]. In this technique, powders from poly(ethylene oxide) (PEO) and PCL were used as the base starting materials. Porous 3D objects were created by selectively joining the powders in each layer using a pure polymer solvent as the inkjet printing binder. Synthetic hard tissue regenerative scaffolds were built through the layered printing and bonding procedures. Thinner filaments (200–500 µm in diameter) were obtained by printing polymer solutions rather than using pure polymer solvent as the adhesive binder [82,83]. Later, Giordano et al. reported a dense porous PLA object which can be used as a bone tissue regenerative scaffold through a Waring blender to mill the liquid nitrogen-chilled PLA granules [84]. An Ultra Centrifugal Mill was employed to improve the yield of the starting materials. Theoretically, any materials that can be processed into powders can be used for this 3D printing technology. For the polymer–ceramic mixture, the polymer is usually used as a low melting point binder. A drawback of this technology is that the redundant powder needs to be wiped off after the printing processes. This may lead to some waste and additional procedures. 

Similar to the above-mentioned polymer–ceramic binder jetting technique, Lee and Barlow used a SLS technique to make bioceramic hard tissue engineering scaffolds [85]. Using this SLS technology, porous 3D objects were built by sintering of powdered material on a powder bed with an infrared laser beam focused on a thin layer of the powder, such as HA containing PCL, nylon and wax. When the local particle surface temperature of the powder is raised to the glass transition temperature (i.e., the melting temperature), the powder is melted and results in particle bonding to each other and to the previous layer. A porous 3D object is created by the fused particles being bonded layer-by-layer.

In 1997, Langton et al. developed a user-defined cancellous bone substitute using a STL approach by polymerizing photopolymer resins [86]. Photopolymer resins are mixtures of low-molecular-weight monomers which can be polymerized when activated by special radiant energy, such as ultraviolet laser or masked lamp. This group of technology emerged in 1999 based on the combination of the masked lamp and laser curing photopolymerization techniques. Since then the preparation of customized hard tissue and organ structural models and/or substitutes has become more and more popular. 

In 1998, Chu et al. built another HA-based prototype for producing bone tissue engineering scaffolds from image-based design files [87]. This ceramic bone tissue engineering scaffolds are created using a UV-curable suspension of HA powders in acrylates. Viscosity control for the highly concentrated HA suspensions and cure depth behavior are the main issues of this technique. Meanwhile, Steidle et al. fabricated a non-resorbable bioceramic bone repair scaffold, which consisted of HA particles and a calcium phosphate glass using a LOM technology [88]. Molecular Geodesics, Inc. (MGI, Boston, MA, USA) developed a new class of hard tissue engineering substitutes that mimic the structural, mechanical and biological characters of the ECMs of the hard tissues. A small-spot laser STL system was used to produce a smallest structural feature of 70 µm in diameter of the printed filaments [89]. 

In 2003, a group in the School of Mechanical and Aerospace Engineering, Nanyang Technological University, Singapore, led by professor Chua, developed a 3D printing technique for customized scaffold fabrication with controlled go-through pore sizes and topological structures [90]. Later they made a collagen scaffold using an indirect 3D printing technique [45].

The above mentioned primary extrusion-based low-temperature 3D printing technology developed at Tsinghua University in 2000 has been mainly used for hard tissue engineering scaffold manufacturing [61,62,63,64,65,66,67,68]. Synthetic biodegradable polymers, such as poly(l-lactic acid) (PLLA) and PLGA, have been fabricated into large 3D bone repair scaffolds under the temperature below −20 °C [2,3]. Some inorganic additives, such as HA and tricalcium phosphate, were incorporated in the polymer solution to increase the mechanical strengths of the scaffolds and mimic the components of the ECMs of the hard tissues. One drawback of this technique is that the synthetic polymers need to be dissolved in organic solvents before printing. The organic solvents need to be removed from the scaffolds throug freeze-drying.

### 3.2. Construction of Patient-Specific Tissues

With the help of 3D printing technologies, customized or patient-specific tissues are now available for hard tissue and organ engineering. An important aspect in the production of customized tissues using 3D printing technologies is to generate digital models for the implants and harvest autologous cells from the patients. Autologous cells are obtained from the same individual in whom they will be implanted to avoid immune rejection. The digital models can be calculated by mirroring a healthy tissue or organ on the corresponding defect area and subsequently transferring and simplifying the data. Based on the 3D obtained data, patient-specific implants, including autologous cell-laden polymer hydrogels, can be prepared using one or several of the above mentioned 3D bioprinting approaches [91,92,93,94,95].

For large skull, oral and maxillofacial surgery, the individual shape of the implants for reconstruction for the original function and aesthetics is required. Patient medical data has to be analyzed and implemented to create predefined standard geometries. For this reason, the large defects of the patient are necessary to be scanned with CT technique before a 3D printing technology is employed. The resulting two-dimensional (2D) data are converted into a 3D surface model with the aid of a special segmentation software. The individual 2D and 3D regions are, thereby, distinguished by the selection of the corresponding threshold value for the segmentation [96,97].

Progress in this field has been extremely rapid. For instance, at the beginning, the CT scanning technique was employed only for the purpose of getting a digital model of the damaged tissues and organs. RP was primarily introduced into this field as a means of guiding surgical procedures. The first customized titanium orbital implant was built around 2001 using a tactile model derived from the patient CT data [91,92,93,94,95]. With the rapid development of 3D bioprinting technologies, now patient-specific tissues, generated through the CAD digital models derived from the CT results, including multiple autologous cell types, hard tissue ECMs or even metal constitutes can be directly used for ideal clinical repairs [35]. An obvious benefit of the patient-specific tissues is that their mechanical properties are similar to those of the natural bones. Unlike traditional tissue engineering strategies, it is not obligatory for the metal constitutes to be biodegraded quickly in the body. However, some side reactions, such as electric conduction, ion exudation and liquid corrosion, need to be clearly considered for each patient before implantation. In some locations of the hard tissues and organs these side reactions have no adverse effects on the hard tissue and organ repair and functionality. An additional benefit of the metal constitutes is that in those areas where bending stiffness and strength are required, the metal constitutes can be compacted. When host tissue grows into the printed go-through channels, the metal scaffolds can tightly integrate into the body tissues. This is a totally new strategy for traditional tissue engineering approaches. 

Currently, there is a high clinical need for novel biological implants that are made of biodegradable synthetic polymers, autologous cells and/or growth factors for patient-specific hard tissue and organ engineering. In other words, synthetic biological hard tissue and organ substitutes are increasingly demanded by medical personnel. Many kinds of 3D printing technologies have been applied to the patient-specific large hard tissue and organ engineering. On the one hand, the ideal synthetic ECMs need to be adapted to the large defect site of the patient to enable an ideal reconstruction. On the other hand, autologous cells and growth factors need to be incorporated into the implants before implantation. Ideally, the large defects can be repaired with newborn tissues during the synthetic ECMs degrade in the same time period. 

### 3.3. Hard Organ Printing 

In addition to producing scaffolds for hard tissue engineering, 3D printing technology is also used to create multiple cell-laden constructs for hard organ engineering [98]. The multiple cell type printing can overcome some of the limitations of conventional scaffold based tissue engineering approaches, such as uneven cell seeding in the scaffolds, dead core in the thick tissues, difficulty in multiple cell incorporation and unable to create uniaxial branched vascular and/or nervous networks in a construct. The available protocols for complex organ manufacturing are absolutely different from the traditional tissue engineering approaches with respect to biological, mechanical, structural and/or biochemical aspects. 

In 2003, Boland et al. printed cells into a virtual 3D structure using a thermal inkjet printing technique. Since then the concept of cell printing has been expanded rapidly from cells to tissues and to organs with several papers and a burst of conduct literature [99,100,101,102,103]. However, a significant limitation of the inkjet-based 3D bioprinting technology is that the shear force from the rapid printing irreversibly damages the cells. Additionally, the most useful 3D structures in this technology are electrical objects, in which the printing is closely related to the material hydrodynamics and support structures [104]. Until now, only simple 3D cell-laden constructs have been produced using cell suspensions or aggregations with limited height and material constitutes. 

Universally, most of the 3D bioprinting technology is initially used for soft tissue and organ (e.g., the liver, heart and kidney) engineering. With the addition of hard inorganic materials, such as HA and calcium phosphate, nearly all of them have been adapted for hard tissue and organ analog (such as the bone, nose, ear, and tooth) manufacturing [105,106,107,108,109]. Recently, there has been a trend towards the utilization of autologous stem cells, such as adipose-derived stem cells (ADSCs) and induced pluripotent stem cells (iPSCs), from the patients (such as, bone marrow and adipose tissues) for organ printing. Multiple nozzle (or multi-nozzle) 3D printers have been employed to assemble the multiple autologous cells, growth factors and other bioactive agents. Normally, for a large structural organ engineering, a larger number of cells are needed. For a large vascular organ engineering, stem cells and growth factors are good candidates for potential proliferation and differentiation capabilities. It has been found that many stem cells are capable to be differentiated into a variety of tissue types, including bone, cartilage and tooth, before or after 3D bioprinting [110,111,112,113,114,115]. 

In the 3D organ printing field, extrusion-based technologies have become increasingly important based on the following reasons: (1) compared with inkjet- and laser-based printing technologies, it is much easier for the hardware and software to be updated; (2) new printers are relatively ready to be designed; (3) multiple cell types can be obviously convenient incorporated; (4) large scale-up structures can be achieved simply through adjusting the printing parameters; (5) costs are relatively low; and (6) using combined multi-nozzle 3D printers, it is possible to overcome nearly all the problems that are encountered by tissue engineering in organ manufacturing experienced in the past (Table 1) [116,117,118,119,120,121,122,123,124,125,126,127,128,129,130,131,132,133,134,135,136,137,138,139,140,141,142,143,144].

**Figure 6:**
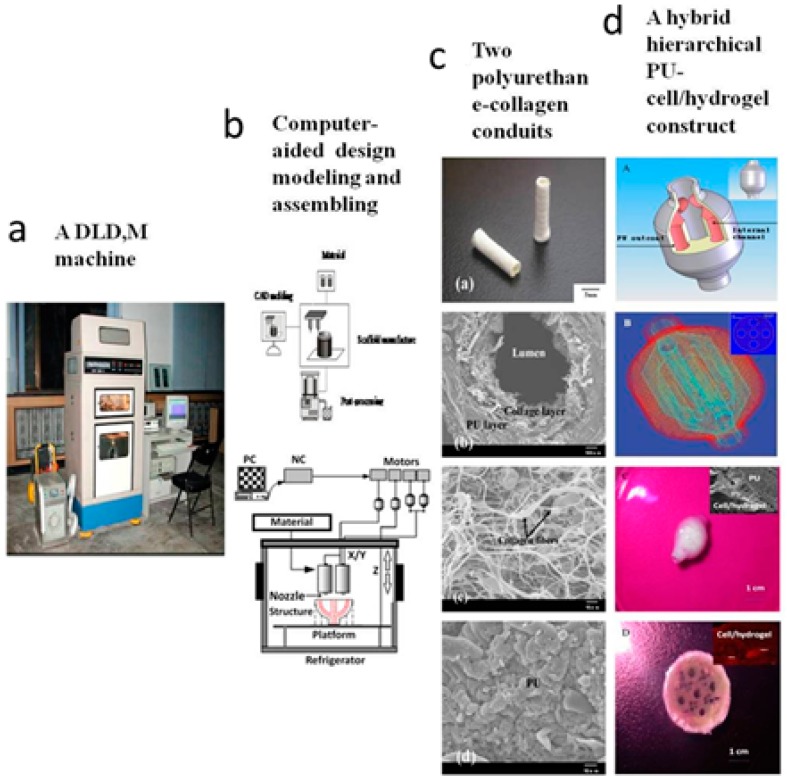
A double-nozzle low-temperature (DLDM) technology developed at Tsinghua University, prof. Wang’ group: (**a**) the DLDM printer; (**b**) schematic description of the working processes of the two nozzles; (**c**) a tubular polyurethane-collagen conduit made by the DLDM system; and (**d**) an elliptical hybrid hierarchical polyurethane and cell/hydrogel construct made by the DLDM system [12].

As an outstanding example, Professor Wang and her students at the Center of Organ Manufacturing and Department of Mechanical Engineering, Tsinghua University, China, have made a series of unique extrusion-based 3D printing technologies for various tissue and organ manufacturing (Figure 6) [12,13,14,15,16,17,18,19,20,21,22,23,116,117,118,119,120,121,122,123,124,125,126,127,128,129,130,131,132,133,134,135,136,137,138,139,140,141,142,143,144]. In the extrusion-based cell, tissue and organ printing technologies, gelatin-based natural polymers are dissolved in inorganic solvents, such as cell culture medium, to form solutions or hydrogels with high viscosity. After the cells were mixed with the natural polymer solutions or hydrogels, they were reversibly encapsulated and allowed to be printed layer-by-layer with a piston-driven extrusion-based 3D printer [12,13,14,15,16,17,18,19,20,21,22,23,116,117,118,119,120,121,122,123,124,125,126,127,128,129,130,131,132,133,134,135,136,137,138,139,140,141,142,143,144]. Both physical and chemical crosslinks are necessary for the integrity maintenance of the 3D printed cell-laden structures. This is due to the gelatin-based hydrogel state is very dependent on temperature. Above 30 °C, the physical crosslinking of the gelatin-based hydrogel is broken and the structural integrity of the printed 3D structure collapses. Long-term in vitro cultures of the 3D structures in culture medium can lead to some of the chemical crosslinks loses. Some new synthetic polymers, such as PU, with excellent biocompatibilities and mechanical properties have been used for the vascular system enhancement and whole structural stabilization overcoat [12,13,14,15,16,17,18,19,20,21,22,23,116,117,118,119,120,121,122,123,124,125,126,127,128,129,130,131,132,133,134,135,136,137,138,139,140,141,142,143,144].

Subsequently, this group has been the leader towards the goal of complex organ manufacturing with a great of significant breakthroughs over the last decade [12,13,14,15,16,17,18,19,20,21,22,23,118,119,120,121,122,123,124,125,126,127,128,129,130,131,132,133,134,135,136,137,138,139,140,141,142,143,144]. For example, it was the first time that cells encapsulated in biodegradable hydrogels (such as gelatin-based hydrogels) were used to print large scale-up 3D structures [69]; as well as the multiple steps of polymer crosslinking and cocktail stem cell engagement in a 3D printed structure [70,71]. They developed the first 3D bioprinted structures that are used for energy model establishment and high throughput drug screening [72,73]. In addition, ADSCs in a grid 3D printed structure were induced into both endothelial and adipose tissues with the spatial effects and made the visualization of the large vascular tissues (i.e., organs) manufacturing come true [72,73]. The two nozzle extrusion-based 3D bioprinting technologies were developed in 2007 to manufacture bioartificial organs with more than two types of cells [74,75]. It was also the first report that multiple cell types, such as ADSCs and hepatocytes, assembled into vascular liver tissues with a uniaxial branched vascular system [124,125]. Later in 2009, the 3D bioprinting techniques were combined with cell cryopreservation techniques successfully to store and preserve the bioartificial tissues and organs [120,121,122]; at the meantime, two level gradient tumor bone repair materials were developed using their home-made double-nozzle low-temperature 3D printing technology [64]; it was the first report that natural and synthetic polymer systems were printed into hierarchical constructs with a predesigned vascular template for in vivo implantation [66,67,68]. The printing cells could be any types, including osteoblasts and chondrocytes. Furthermore, they also first developed a series of combined multi-nozzle 3D bioprinting technologies for complex hard tissue and organ engineering. The above technologies were printed in the first book on *Organ Manufacturing* published in America by the Nova Science Publishers Inc., Hauppauge, NY, USA [18,19,21,23]. Some of the above technologies have been adopted by many other research groups, such as the Wake Forest Institute for Regenerative Medicine, Wake Forest School of Medicine, Medical Center Boulevard, Winston-Salem, NC, USA, for cartilage and ear manufacturing [35].

Particularly, the establishment of the scale-up hierarchical vascular system was a long awaited breakthrough in tissue engineering, organ manufacturing and regenerative medicine [72,73,134,139,141]. These overwhelming developments in multi-nozzle bioprinting technologies yielded a novel set of organ regeneration strategies. The in-depth studies about complex organ manufacturing were accompanied with a series of new theories and protocols, such as a combined multi-nozzle 3D printer is essential for vascular organ manufacturing; both natural and synthetic polymers are useful to provide heterogeneous cells with a suitable environment to survive, proliferate, and differentiate in a construct; the natural polymers need to be double crosslinked to maintain the structural integrity of the printed 3D cell-laden structures; the weak mechanical properties of natural polymer hydrogels can be compensated by the synthetic polymers; spatial effect of stem cell engagement is necessary for a whole vascular system generation; the soft organ 3D printing technologies are also suitable for complex hard tissue and organ engineering with the incorporation of particular inorganic ECMs, cell types and/or growth factors in the natural or synthetic polymer solutions [35]. It is expected that the complex organ manufacturing era is finally coming and the average life span of human beings will be extremely prolonged with these series of outstanding breakthroughs. 

## 4. Conclusions and Future Directions

Modern 3D printing technology has enabled the production of hard tissue and organ 3D models, scaffolds and analogs directly from CAD data. The 3D printing technology can be classed into various categories according to different techniques. Especially in the field of surgical technology these technologies were used for the production of patient-specific implants, such as porous scaffolds, cell-laden constructs, bioartificial tissues and organs, for hard tissue and organ engineering. The integration of CT techniques, visual hard tissue and organ regenerative models and biological functional prototypes has attained great successes in large tissue and organ defect healing and repair. Over the last decade, the extrusion-based 3D printing technologies have developed very quickly and outstood among all the available protocols. The inkjet- and laser-based 3D printing technologies are in the second or third location due to the limitations of software and hardware of the inkjet-based equipment and the time-consuming and damage effects to cells of the laser-based devices. Recognition should be given to the low-temperature double-nozzle extrusion-based 3D printing technologies, which are especially useful for patient-specific hard tissue and organ engineering. A prominent accomplishment is the uniaxial branched vascular system incorporated in the large tissues and organs. Many major problems for vascular organ engineering were overcome by the Wang Group in the Center of Organ Manufacturing, Department of Mechanical Engineering, Tsinghua University. Compared with traditional tissue engineering strategies, in which cells need to be seeded onto porous scaffolds to form tissues, their in house 3D bioprinting technology has many advantages in creating bioartificial tissues and organs. The gelatin-based natural hydrogels and synthetic polymers have provided the cells with a compliant accommodation to grow, proliferate and differentiate. The controlled go-through channels and branched vascular systems are essential for blood/body fluid passing through/penetration and vascularization/new tissue in-growth. Their scientific advances in extrusion-based combined multi-nozzle 3D bioprinting technology, novel PU biomaterials, stem cell engagement protocols, bioactive agent (e.g., cryoprotectant, growth and differentiation factor) incorporation techniques, and spatial macro- and micro-environment controlling strategies give rise to opportunities for manufacturing bioartificial hard tissues and organs with the whole spectrum of their native counterparts.

Currently, much more meaningful research platforms and 3D bioprinting tools on the one hand are being exploited for hard tissue and organ engineering. On the other hand, reverse hard tissue and organ engineering from patient-specific CT and MRU data have attracted more and more attention for the high frequent traffic accidents and lumpectomies. It is expected that in the future, custom tissue and organ banks and patient-specific hard tissue and organ repairs will become prevalent. Especially, combined multi-nozzle bioprinting technology will be the most important tools for complex hard tissue and organ engineering. The combined multi-nozzle bioprinting technology has distinct advantages in producing complex tissue and organ substitutes mimicking their native counterparts with a predesigned branched vascular network. Multiple cell types together with heterogeneous biochemical molecules (i.e., biological cues, bioactive agents or drugs) can be precisely constructed through the combined multi-nozzle 3D printers. Stem cells, progenitor cells and decellularized ECMs will become more and more popular for a large tissue and organ printing. There is a need for sustained release of growth factors and other bioactive agents over time. With the advances of modern 3D bioprinting sciences and technologies, the era of patient-specific hard tissue and organ manufacturing is coming and clinical standards for the bioartificial hard tissues and organs will be a common subject in hospitals.

## Figures and Tables

**Table 1:** Typical three-dimensional (3D) bioprinting technologies for hard tissue and organ engineering.

Technique	Working principle	Main starting biomaterials	Advantages	Disadvantages	Morphology	References
Extrusion-based rapid prototyping (RP)	Fluidic material is forced through a piston nozzle at a low temperature (≤−20 °C)	Natural or synthetic polymer solutions	A wide range of materials can be used; high accuracy; flexible; reproducible; scalable; growth factors can be incorporated; constructs with high mechanical properties can be obtained	Organic solvents are needed for synthetic polymer deposition; cells are difficult to be incorporated	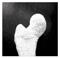	[59]
Pneumatic extrusion-based bioplotter	Polymer strands stabilized layer-by-layer in a liquid medium	Natural polymer solutions, such as alginate and proteins, cells and growth factors can be incorporated	Good biocompatibilities	Low cell survival rate; weak mechanical properties; fragile	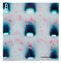	[141]
Fused deposition modeling (FDM)	Strands of heated polymers extruded through nozzles	Synthetic polymers, such as acrylonitrile butadiene styrene (ABS), poly lactic acid (PLA), polyvinyl alcohol (PVA)	Automated; controllable; fast; sophisticated; accurate; reproducible; scalable	Limited materials can be used; cells cannot be incorporated directly	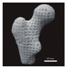	[142]
FDM	Strands of polymer composite extruded through a commercial FDM (MakerBot)	Hydroxyapatite (HA) incorporated polycaprolactone (PCL)	Automated; controllable; fast; sophisticated; accurate; reproducible; scalable	Limited materials can be used; cells cannot be incorporated directly	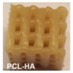	[143]
Indirect 3D bio-printing	Fibrin-polymer–ceramic scaffolds manufactured by fused deposition modeling	Calcium phosphate modified PCL (PCL-CaP) and treated with fibrinogen	A wide range of biomaterials can be used; cells and bioactive agents can be incorporated	Low accuracy of the final structures; complex processing procedures	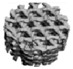	[144]
Indirect micro-stereolithography (mSTL)	Tracheal cartilage regeneration on an indirect printed gelatin sponge	Poly-(l-Lactide-*co*-ε-caprolactone)/gelatin, heparin, transforming growth factor-*β*1, chondrocytes	A wide range of biomaterials can be used; bioactive agents can be incorporated	Low accuracy of the final structures; complex processing procedures; limited mechanical properties	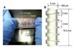	[111]
Laser-based stereolithography (SLA)	A small-spot of laser is used for solid polymers	Synthetic polymers	High resolution; cells can be incorporated	Limited materials; low throughput	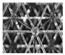	[54,85]
Thermal inkjet-based AM	Collagen was dissolved into phosphoric acid-based binder solution to fabricate collagen-calcium phosphate composites	Collagen solutions	The fabrication temperature can be reduced	Low accuracy; low mechanical properties; cells cannot be incorporated	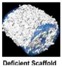	[113]
Extrusion-based RP	Pneumatic forced nozzles for fluidic materials	Natural or synthetic polymer solutions	A wide range of biomaterials can be used; cells, bioactive agents can be incorporated	Nozzle easily clogging; harms to cells	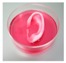	[35]
Inkjet-based RP	Fluidic material is forced through an orifice	Hyaluronic acid (HA) improved gelatin-methacrylamide (gelMA) hydrogels	High mechanical properties; cells, bioactive agents can be incorporated	Limited biomaterials can be used; limited height of the construct	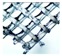	[144]
Direct write (DW) RP	3D ink writing (or robocasting) in an oil bath	A concentrated colloidal gel (typically 50% HA particles suspended in an aqueous medium)	Two materials can be printed in a construct	Limited biomaterials can be used; limited height of the construct	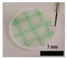	[95]
Double nozzle extrusion-based RP	Fluidic materials are forced through two piston nozzles at a temperature about 10 °C	Natural polymer hydrogels, such as gelatin, gelatin/alginate, and gelatin/alginate/fibrinogen	A wide range of biomaterials can be used; cells, bioactive agents can be incorporated; branched vascular systems can be easily created; excellent biocompatibilities	Weak mechanical properties; high concentration of hydrogels affects cell–cell interactions; easily being biodegraded under in vivo conditions	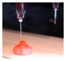	[120,121]
Double nozzle low-temperature extrusion-based RP	Fluidic materials are forced through two piston nozzles at a temperature ≤−20 °C	Natural and synthetic polymer solutions	A wide range of biomaterials can be used; cells, growth factors, cytokines, chemicals, genes can be incorporated; branched vascular systems can be easily created; high mechanical properties; stable; fast; controllable; sophisticated; accurate; scalable; reproducible	High concentration of natural hydrogels affects cell–cell interactions; organic solvents are needed for synthetic polymer dissolution and to be removed after printing	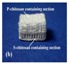	[61,62,127,128]
