# Correction: 3D Bioprinting Technologies for Hard Tissue and Organ Engineering. *Materials* 2016, *9*, 802

**DOI:** 10.3390/ma9110911

**Published:** 2016-11-10

**Authors:** Xiaohong Wang, Qiang Ao, Xiaohong Tian, Jun Fan, Yujun Wei, Weijian Hou, Hao Tong, Shuling Bai

**Affiliations:** 1 Department of Tissue Engineering, Center of 3D Printing & Organ Manufacturing, School of Fundamental Sciences, China Medical University (CMU), No. 77 Puhe Road, Shenyang North New Area, Shenyang 110122, China; aoqiang00@163.com (Q.A.); xhtian@cmu.edu.cn (X.T.); jfan@cmu.edu.cn (J.F.); Weiyj2011@gmail.com (Y.W.); wjhou@cmu.edu.cn (W.H.); tongh007@hotmail.com (H.T.); baishuling@hotmail.com (S.B.); 2 Department of Mechanical Engineering, Tsinghua University, Center of Organ Manufacturing, Beijing 100084, China

In the published manuscript “3D Bioprinting Technologies for Hard Tissue and Organ Engineering. *Materials* 2016, *9*, 802 [1]”, a reference (Ozbolat, I.T., Yu, Y. Bioprinting toward organ fabrication: challenges and future trends. *IEEE Trans. Biomed. Eng.* 2013, *60*, 691–699) has been omitted from the references. Alterations are therefore requested to add this reference before the original reference 22 in the reference list, in the legend of Figure 2, as well as in the context (such as [17–23] in page 2 line 15, [12–23,26–28] in page 2 line 23). The following references after reference 22 therefore need to be changed. We apologize for the inconvenience this will have caused. In addition, the following changes to the text have been made:

Page 11, Section 3.3, the expression “such as stem cells” which appears six lines up from the bottom should be changed to “such as adipose-derived stem cells (ADSCs)”.

Page 18, reference 46, the page range should be “298–310”.

The changes do not affect the results. The manuscript will be updated and the original will remain online on the article webpage.
